# Trichodimerol inhibits inflammation through suppression of the nuclear transcription factor-kappaB/NOD-like receptor thermal protein domain associated protein 3 signaling pathway

**DOI:** 10.3389/fmicb.2022.999996

**Published:** 2022-08-23

**Authors:** Xue-Yan Huo, Li-Rong Lei, Wen-Xiu Guo, Yun-Jie Hu, Qi-Xuan Kuang, Meng-Dan Liu, Wan Peng, Yi-Fei Dai, Dong Wang, Yu-Cheng Gu, Da-Le Guo, Yun Deng

**Affiliations:** ^1^State Key Laboratory of Southwestern Chinese Medicine Resources, School of Pharmacy, Chengdu University of Traditional Chinese Medicine, Chengdu, China; ^2^Institute of Rare Diseases, West China Hospital of Sichuan University, Chengdu, China; ^3^Department of Basic Medical Sciences, School of Medicine, Tsinghua University, Beijing, China; ^4^Syngenta Jealott’s Hill International Research Centre, Berkshire, United Kingdom

**Keywords:** trichodimerol, inflammation, NF-κB, NLRP3, molecular docking

## Abstract

Excessive inflammation causes chronic diseases and tissue damage. Although there has been drug treatment, its side effects are relatively large. Searching for effective anti-inflammatory drugs from natural products has become the focus of attention. First isolated from *Trichoderma longibraciatum*, trichodimerol is a natural product with TNF inhibition. In this study, lipopolysaccharide (LPS)-induced RAW264.7 macrophages were used as a model to investigate the anti-inflammatory activity of trichodimerol. The results of nitric oxide (NO) detection, enzyme-linked immunosorbent assay (ELISA), and reactive oxygen species (ROS) showed that trichodimerol could reduce the production of NO, ROS, and the proinflammatory cytokines interleukin (IL)-6 and tumor necrosis factor (TNF)-α. Western blotting results showed that trichodimerol could inhibit the production of inflammatory mediators such as cyclooxygenase (COX)-2 and inducible nitric oxide synthase (iNOS) and the protein expression of nuclear transcription factor-kappaB (NF-κB), p-IKK, p-IκB, Toll-like receptor 4 (TLR4), NOD-like receptor thermal protein domain associated protein 3 (NLRP3), cysteinyl aspartate specific proteinase (Caspase)-1, and ASC, which indicated that trichodimerol may inhibit inflammation through the NF-κB and NLRP3 pathways. At the same time, molecular docking showed that trichodimerol can directly combine with the TLR4-MD2 complex. Hence, trichodimerol inhibits inflammation by obstructing the interaction between LPS and the TLR4-MD2 heterodimer and suppressing the downstream NF-κB and NLRP3 pathways.

## Introduction

Excessive inflammation can lead to a series of chronic diseases and tissue damage. Due to the side effects of marketed drugs, the search for new efficacious and safe anti-inflammatory natural products with novel structures is still a focus of extensive research (Zhong and Shi, 2019). Fungi are highly rewarding resources of auspicious hit compounds for inflammation-related diseases, and many anti-inflammatory natural products with novel, complex, and compact structures have been isolated from fungi (Cao et al., 2021; Ju et al., 2021; Huang et al., 2022; Kuang et al., 2022a,b). Trichodimerol is a typical natural product isolated from *Trichoderma longibraciatum*. It has been reported that trichodimerol can inhibit the secretion of proinflammatory factors, including tumor necrosis factor (TNF-α) and nitric oxide (NO; Lee et al., 2005). However, the underlying mechanism is currently unclear.

In this study, lipopolysaccharide (LPS)-induced RAW264.7 macrophages and zebrafish were used to investigate the anti-inflammatory activity and reveal the related underlying mechanism of trichodimerol. The results showed that trichodimerol reduced the production of NO, ROS, and the proinflammatory cytokines interleukin (IL)-6 and TNF-α. Western blotting results also indicated that trichodimerol could inhibit the production of inflammatory mediators such as cyclooxygenase (COX)-2 and inducible nitric oxide synthase (iNOS) and the protein expression of nuclear transcription factor-kappa B (NF-κB), p-IKK, p-IκB, Toll-like receptor 4 (TLR4), NOD-like receptor thermal protein domain associated protein 3 (NLRP3), cysteinyl aspartate specific proteinase (Caspase)-1 and ASC, which indicated that trichodimerol may inhibit inflammation through the NF-κB and NLRP3 pathways. In addition, molecular docking indicated that TLR4 was directly combined with trichodimerol, which can provide an interpretation of the inhibition of the NF-κB and NLRP3 pathways. The details of the anti-inflammatory activity and partial underlying mechanisms of trichodimerol are reported herein.

## Materials and methods

### Materials

Fetal bovine serum (FBS) was purchased from Excell (FCS500, United States). Dulbecco’s modified Eagle’s medium (DMEM) was purchased from Gibco (C11995500BT, United States). Penicillin–streptomycin was purchased from HyClone (SV30010, United States). Phosphate buffered saline (PBS) was purchased from Boster (AR0030, Wuhan, China). Dimethyl sulfoxide (DMSO) was purchased from Gibco. Lipopolysaccharide (LPS) was purchased from Beyotime (ST1470, Shanghai, China). Radioimmunoprecipitation assay buffer (RIPA) was purchased from Beyotime (P0013B, Shanghai, China). Broad spectrum protease inhibitor cocktail and broad phosphatase inhibitor were purchased from Boster (Wuhan, China). Cell Counting Kit-8 reagent (CCK8) was purchased from MCE (HY-K0301, United States). Total RNA extraction reagent was purchased from Vazyme (R401-01, Nanjing, China). A BCA Protein Assay Kit was purchased from CWBIO (CW0014S, Beijing, China). The PAGE Gel Rapid Preparation Kit was purchased from Yamei (PG112, Shanghai, China). Omni-Easy™ Protein Sample Loading Buffer was purchased from Yamei (LT101S, Shanghai, China). The Nitric Oxide (NO) Assay Kit was purchased from Beyotime (S0021S, Shanghai, China). The Mouse TNF-α ELISA Kit was purchased from Boster (EK0527, Wuhan, China). The Mouse IL-6 ELISA Kit was purchased from Boster (EK0411, Wuhan, China). RT EasyTM II (Master Premix for first-strand cDNA synthesis for Real-Time PCR RT-01022) and Real-Time PCR EasyTM-SYBR Green I (QP-01012) were purchased from Foregene (Chengdu, China). A Reactive Oxygen Species Assay Kit was purchased from UElandy (R6033, Suzhou, China). The NF-κB Activation, Nuclear Translocation Assay Kit (rabbit polyclonal antibody) was purchased from Beyotime (SN368, Shanghai, China). A 180 kDa Prestained Protein Marker was purchased from Vazyme (MP102-02, Nanjing, China). Western Blocking Buffer was purchased from Beyotime (P0023B, Shanghai, China). Super ECL Plus Western Blotting Substrate was purchased from Biogeound (BG0001, Chongqing, China).

### Cell culture

RAW264.7 macrophages were cultured in DMEM supplemented with 10% fetal bovine serum and 1% penicillin and streptomycin antibody, and the living environment was 37 °C incubator containing 5% CO_2._

### Cell viability

RAW264.7 macrophages were cultured on 96-well plate with 1,000 cells per well. After 6 h, different concentrations (3.75, 7.5, 15, 30, 60, 120, and 240 μM) of trichodimerol mixed in the culture medium were added to the 96-well plate for incubation for 48 h. Afterward, 10 μl of CCK8 was added to each well for 1 h. The number of cells was detected by enzyme calibration at a wavelength of 450 nm.

### Determination of NO production

RAW264.7 macrophages were cultured on 6-well plate with 10,000 cells per well overnight and then pretreated with simple trichodimerol and different concentrations of trichodimerol (5, 10, and 15 μM) for 2 h with LPS (1 μg/ml) added for 24 h. DMSO was used as a negative control. Then, the supernatant was absorbed, and the Griess reagent system was used to detect NO production.

### Reverse transcription-PCR analysis

RAW264.7 macrophages were plated on six-well plate with 100,000 cells per well. After 24 h, the cells were pretreated with simple trichodimerol and different concentrations of trichodimerol (5, 10, and 15 μM) for 2 h and then with LPS (1 μg/ml) for 24 h. Then, 1 ml of TRIzol reagent was added to extract RNA. RNA purity and concentration were measured with an ultramicrospectrophotometer. After that, genomic DNA was removed with 4 × gDNA wiper Mix and reverse transcripted with 5 × HiScriptIIqRT SuperMixII, cDNA amplification was carried out with the ChamQ Universal SYBR qPCR Master Mix, in which the 2 × ChamQ Universal SYBR qPCR Master Mix was 5 μl, the DNase-free ddH_2_O was 2.1 μl, the Template cDNA was 2.5 μl and the primers COX-2 (forward primer: 5′-AACCCAGGGGATCGAGTGT-3′, reverse primer: 5′-CGCAGCTCAGTGTTTGGGAT-3′), iNOS (forward primer: 5′-GAGCCACAGTCCTCTTTGCTA-3′, reverse primer: 5’-TGTCACCACCAGCAGTAGTTG-3′), IL-1β (forward primer: 5′-TGAAATGCCACCTTTTGACAG-3′, reverse primer: 5′-CCACAGCCACAATGAGTGATAC-3′), IL-6 (forward primer: 5′-GGGACTGATGCTGGTGACAAC-3′, reverse primer: 5′-CAACTCTTTTCTCATTTCCACGA-3′), TNF-α (forward primer: 5′-CCCTCCAGAAAAGACACCATG-3′, reverse primer: 5′-CACCCCGAAGTTCAGTAGACAG-3′), and GAPDH (forward primer: 5′-GCAAGTTCAACGGCACAG-3′, reverse primer: 5’-CGCCAGTAGACTCCACGAC-3′) was 0.2 μl.

### Western blotting

RAW264.7 macrophages were plated on six-well plate with 100,000 cells per well. After 6 h, the cells were pretreated with simple trichodimerol and different concentrations of trichodimerol (5, 10, and 15 μM) for 2 h and then treated with LPS (1 μg/ml) for 24 h. The cells were removed and washed twice with phosphate buffered saline (PBS). Total protein was extracted by 1 × SDS lysis with 250 μl heated in a constant temperature metal bath at 100°C for 30 min and centrifuged at 12,000 rpm at 4°C for 15 min to obtain the supernatant. The protein concentration was detected by a BCA Protein Assay Kit. Protein sample loading buffer (1×) was used at 95°C for 10 min to prevent denaturation. The total proteins were separated by 10% sodium dodecyl sulfate–polyacrylamide gel electrophoresis (SDS-PAGE), transferred to polyvinylidene fluoride (PVDF) membranes, and incubated with primary antibody at 4°C overnight. The primary antibodies were as follows: TLR4 (1:4,000, Proteintech, 66350-1-Ig, China), NF-κB (1:1,000, CST, 8242S, United States), p-NF-κB (1:1,000, CST, 3033S), p-IκB (1:1,000, CST, 5209S), p-IKKα/β (1:1,000, CST, 2697S), NLRP3 (1:1,000, CST, 15101S), Caspase-1 (1:1,000, Proteintech, 22915-1-Ig), ASC/TMS1 (1:1,000, Proteintech, 69494-1-Ig), iNOS (1:1,000, NOVCCS, NB300-605SS, United States), COX-2 (1:1,000, Abcam, ab179800, United States), GAPDH (1:50,000, Proteintech, 60004-1-Ig), and Tubulin (1:50,000, Proteintech, 66031-1-Ig). Then, the cells were incubated with secondary antibody at room temperature for 2 h. Strips were detected by a high-sensitivity ECL chemiluminescence detection kit and analyzed by ImageJ software.

### Enzyme-linked immunosorbent assay

RAW264.7 macrophages were cultured on six-well plate with 10,000 cells per well overnight and then pretreated with simple trichodimerol and different concentrations of trichodimerol (5, 10, and 15 μM) for 2 h with LPS (1 μg/ml) added for 24 h. Then, the supernatant was absorbed. The inflammatory factors TNF-α and IL-6 were assayed by ELISA kits, and then the absorbance was detected at 450 nm.

### Intracellular ROS measurement

RAW264.7 macrophages were plated on 12-well plate and placed into climbing flasks at a density of 20,000 cells per well. After 6 h, the cells were pretreated with simple trichodimerol and different concentrations of trichodimerol (5, 10, and 15 μM) for 2 h with LPS (1 μg/ml) added for 24 h. The cells were stimulated with DCFH-DA reagent (10 μM) at 37°C for 30 min and washed twice with PBS. The following step was to immobilize with 5% paraformaldehyde for 15 min, wash twice with PBS dye with DAPI for 8 min with paraformaldehyde and wash twice again. Finally, the results were observed under a fluorescence microscope. Zebrafish were pretreated with 2.5 and 5 μM trichodimerol. After 1 h, 10 μg/ml LPS was cultured for 72 h. During this period, fresh trichodimerol and LPS were replaced every 24 h and then treated with DCFH-DA for 1 h and anesthetized with tricaine. Finally, the fluorescence intensity was detected by confocal microscopy (Olympus FV1200, Japan).

### Nuclear transport of NF-κB/p65

RAW264.7 macrophages were plated on 12-well plate at a density of 20,000 cells per well. After 6 h, the cells were pretreated with simple trichodimerol and different concentrations of trichodimerol (5, 10, and 15 μM) for 2 h, then with LPS (1 μg/ml) added for 12 h. In cells, the NF-κB nuclear transport state was treated by the NF-κB Activation, Nuclear Translocation Assay Kit. The operation was as follows: fixation solution was added for 15 min, and the washing solution was washed three times for 5 min each time. After that, the blocking solution was blocked at room temperature for 1 h, and the NF-κB/p65 antibody was incubated at room temperature for 1 h. Then, the washing solution was washed three times for 10 min each time, and the anti-rabbit Cy3 antibody was added at room temperature for 1 h. Finally, the washing solution was washed twice. DAPI staining was performed for 5 min, and the slices were prepared. The results were presented under a fluorescence microscope (Olympus, IX73, Japan).

### Molecular docking

The crystal structure of the TLR4-MD2 complex was obtained from the RCSB protein database (PDB ID: 2Z66; Berman et al., 2000). The docking analysis of trichodimerol and TLR4-MD2 was performed by Schrödinger software. Schrödinger’s Maestro Molecular Modeling Apparatus was used to obtain the 3D structure, regeneration state of natural ligands, crystal structure of protein, optimization of hydrogen bond distribution, energy minimization of trichodimerol, and water removal. Finally, the best binding site was predicted by the SiteMap module (Friesner et al., 2006).

### Statistical analysis

All data were analyzed by GraphPad Prism 7.0 software (San Diego, California, United States) and expressed as the mean ± SD of three repetitions of the same experiment. The data were from three independent experiments.

## Results

### Trichodimerol inhibited LPS-induced inflammation in RAW264.7 macrophages

**Figure 1:**
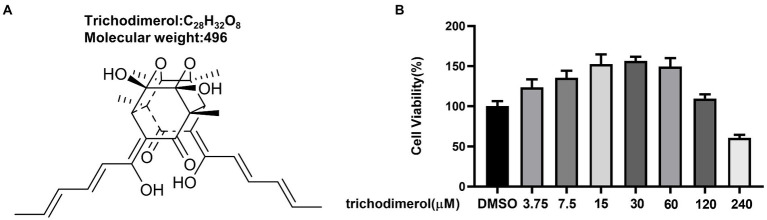
The structure and cytotoxicity of trichodimerol. **(A)** The structure of trichodimerol. **(B)** Cell viability. Cell viability was tested with CCK8 reagent 48 h after RAW264.7 macrophages were administered different amounts of trichodimerol, and dimethyl sulfoxide (DMSO) was used as a blank group.

**Figure 2:**
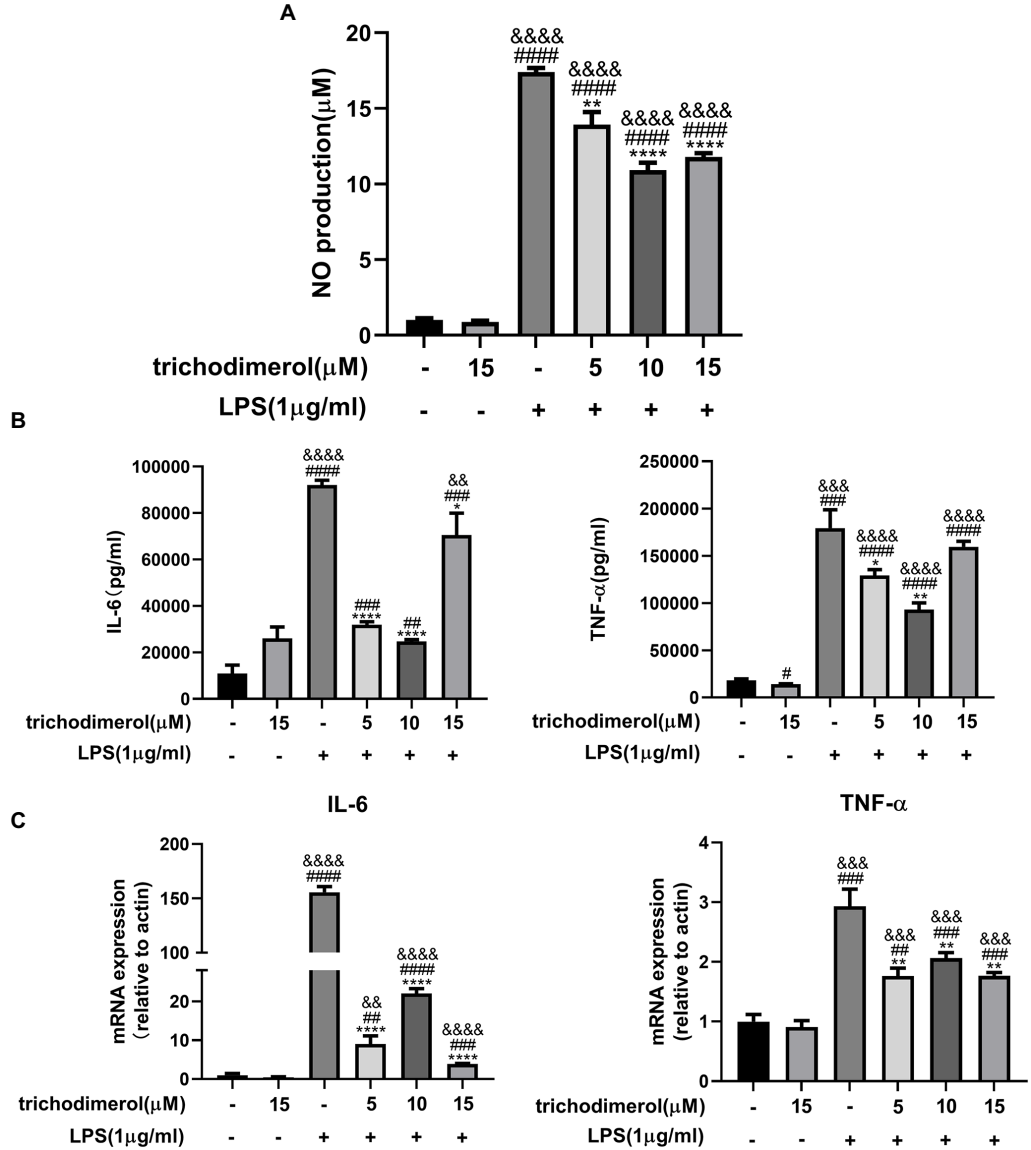
The effect of trichodimerol on inflammation. **(A)** Nitric oxide (NO) production in the supernatant of RAW264.7 macrophages after trichodimerol administration. **(B)** RAW264.7 macrophages were treated with different concentrations of trichodimerol and induced by lipopolysaccharide (LPS). The expression of tumor necrosis factor (TNF)-α and IL-6 in the supernatant was detected by ELISA. **(C)** mRNA expression levels of IL-6 and TNF-α in RAW264.7 macrophages in different treatment groups. All data are expressed as the mean ± SD. ^#^*p* < 0.05, ^##^*p* < 0.01, ^###^*p* < 0.001, and ^####^*p* < 0.0001, compared with the DMSO group. ^&&^*p* < 0.01, ^&&&^*p* < 0.001, and ^&&&&^*p* < 0.0001, compared with the trichodimerol 15 μM group. ^*^p < 0.05, ^**^p < 0.01, and ^****^p < 0.0001, compared with the LPS group.

Trichodimerol was extracted from *Pseudeurotium ovale* (Figure 1A). To study the anti-inflammatory effect of trichodimerol on LPS-induced RAW264.7 macrophages, a cell viability test was performed on the cells. The results showed that the cell viability was better in the range of 120 μM (Figure 1B). Concentrations of 5, 10, and 15 μM were selected for subsequent experiments. RAW264.7 macrophages were treated with trichodimerol and induced by LPS for 24 h, and the supernatant was collected to detect NO and proinflammatory cytokines. The results showed that under the influence of trichodimerol, the release of NO was inhibited (Figure 2A), and the proinflammatory cytokines TNF-α and IL-6 also showed a downward trend, which was consistent with the expression of proinflammatory factor mRNA in Reverse transcription-PCR (RT–PCR; Figures 2B,C).

### Trichodimerol restrained LPS-induced expression or production of inflammatory mediators in RAW264.7 macrophages

**Figure 3:**
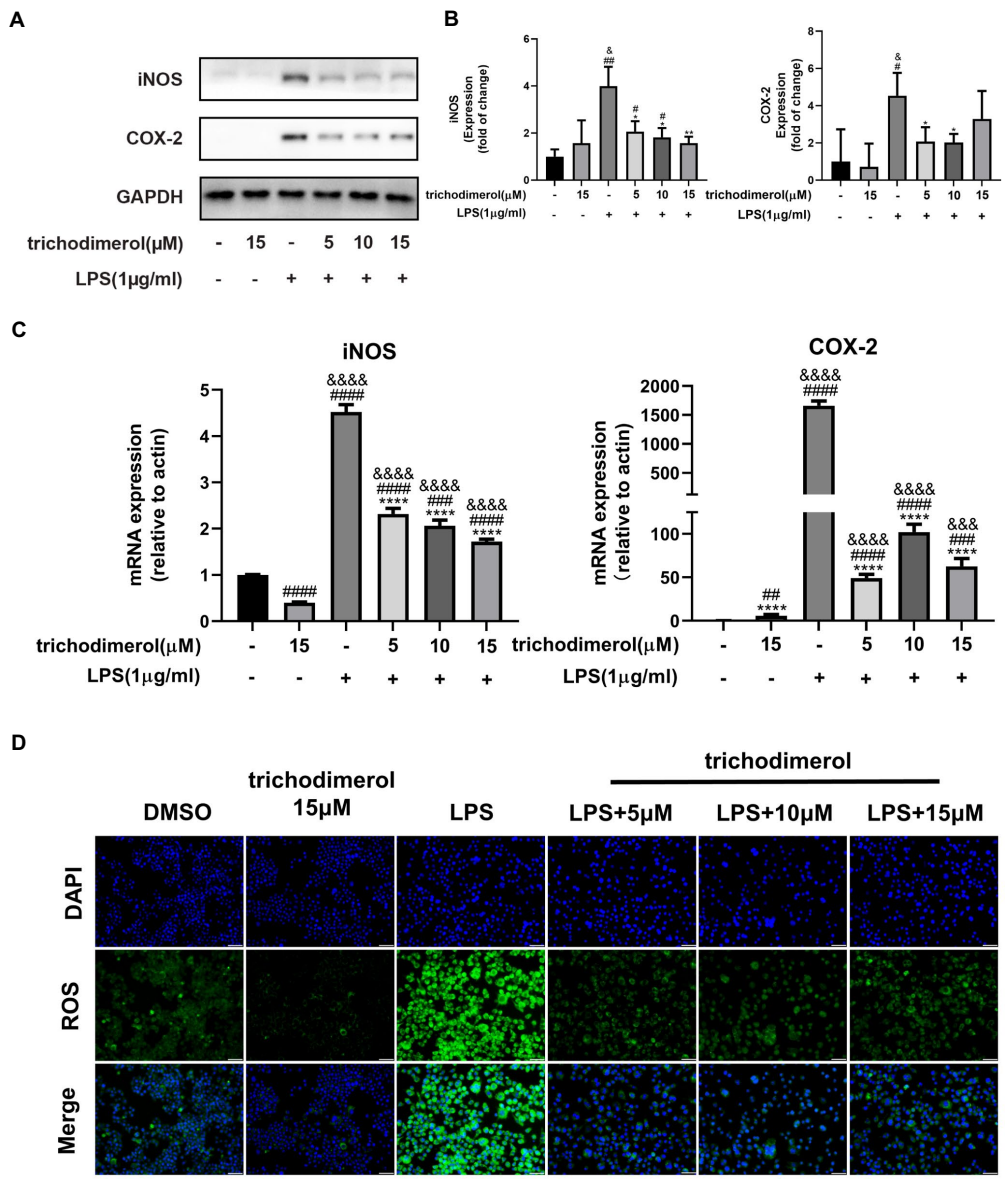
The effect of trichodimerol on the inflammatory mediators. **(A)** The protein expression of inducible nitric oxide synthase (iNOS) and cyclooxygenase (COX)-2 in RAW264.7 macrophages treated with different concentrations; GAPDH was used as an internal reference. **(B)** Quantitative statistical results of the protein expression of iNOS and COX-2. **(C)** mRNA expression levels of iNOS and COX-2 in RAW264.7 macrophages in different treatment groups. **(D)** Reactive oxygen species (ROS) production in RAW264.7 macrophages induced by LPS 24 h after trichodimerol treatment. Green fluorescence represents intracellular ROS stained by DCFH-DA, blue fluorescence represents nucleus, white stripe = 50 μm. All data are expressed as the mean ± SD. ^#^*p* < 0.05, ^##^*p* < 0.01, ^###^*p* < 0.001, and ^####^*p* < 0.0001, compared with the DMSO group. ^&^*p* < 0.05, ^&&&^*p* < 0.001, and ^&&&&^*p* < 0.0001, compared with the trichodimerol 15 μM group. ^*^p < 0.05, ^**^p < 0.01, and ^****^p < 0.0001, compared with the LPS group.

Lipopolysaccharide-induced macrophages overexpress COX-2 and iNOS to increase prostaglandin and NO release (Miletic et al., 2006). Reactive oxygen species (ROS) are mainly generated by mitochondria (Brillo et al., 2021), and excessive release will lead to tissue and organ damage (Yang and Lian, 2020). Western blotting analysis showed that the protein levels of COX-2 and iNOS in LPS-induced RAW264.7 macrophages was significantly decreased after administration of trichodimerol (Figures 3A,B). At the same time, there was the same trend as the expression of inflammatory mediators at the mRNA level in RT–PCR (Figure 3C). Immunofluorescence showed that the green fluorescence intensity decreased after adding trichodimerol, indicating that the release of ROS decreased (Figure 3D).

### Trichodimerol weakened inflammation *in vivo*

**Figure 4:**
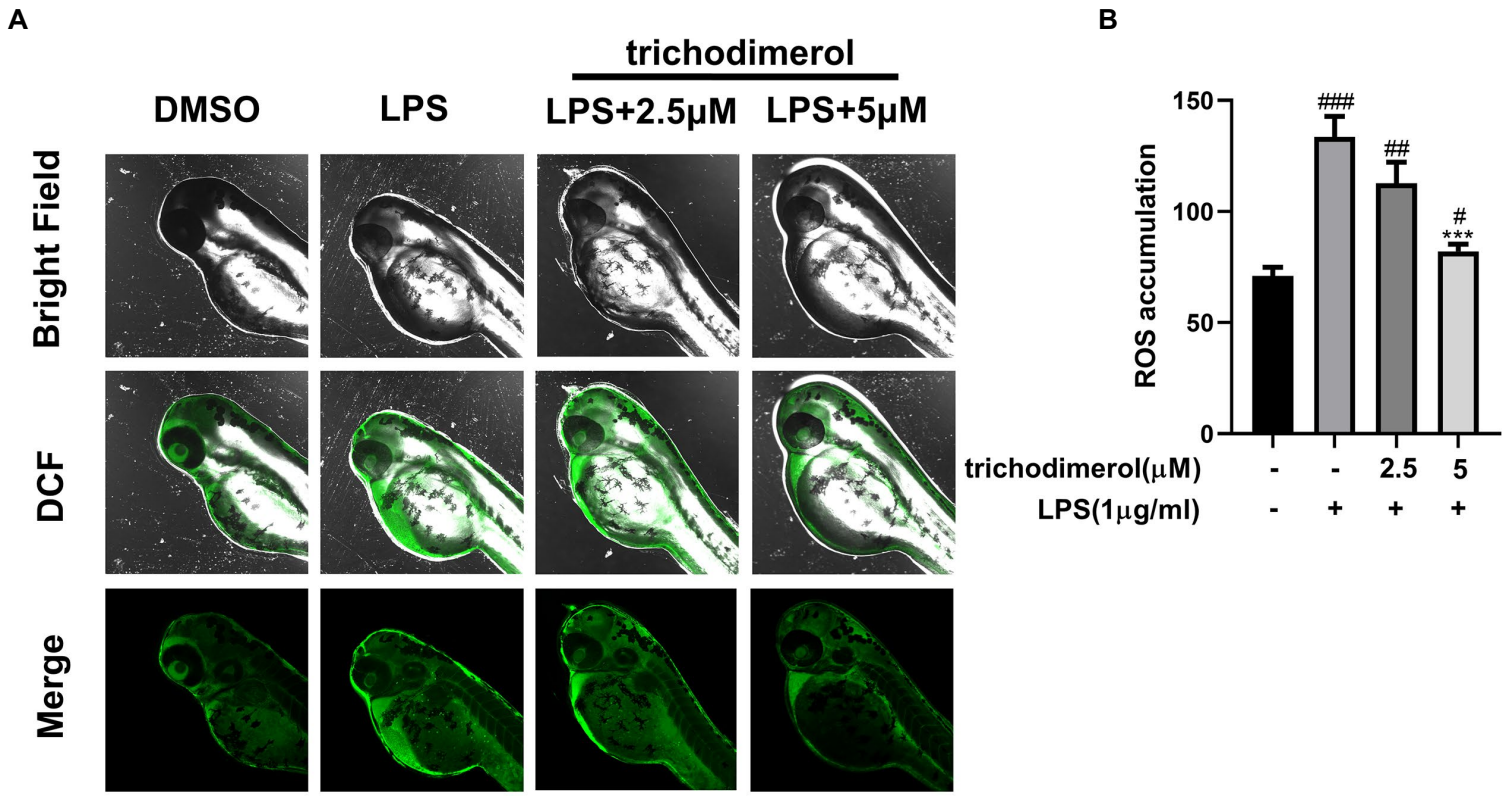
The accumulation of ROS in zebrafish. **(A)** Images of reactive oxygen species (ROS) levels in zebrafish exposed to different amounts of trichodimerol. Green fluorescence represents intracellular ROS stained by DCFH-DA. **(B)** ImageJ was used to quantify the fluorescence intensity, which was statistically analyzed. All data are expressed as the mean ± SD. ^#^*p* < 0.05, ^##^*p* < 0.01, and ^###^*p* < 0.001, compared with the DMSO group. ^***^p < 0.001, compared with the LPS group.

Zebrafish is a significant model system for analyzing human diseases. Studies have found that the zebrafish genome shares 60–80% homology with the human genome (Barbazuk et al., 2000). Zebrafish have the advantages of strong reproduction, fast development, and small size (Jia et al., 2019). Currently, an increasing number of zebrafish have been used in the *in vivo* study of inflammatory animals (Zanandrea et al., 2020). In this experiment, the green fluorescence intensity of zebrafish was significantly downregulated compared with that of the LPS group (Figures 4A,B), indicating that trichodimerol inhibited the release of ROS, which is consistent with the *in vitro* experiment.

### Trichodimerol inhibited the NLRP3 pathway

**Figure 5:**
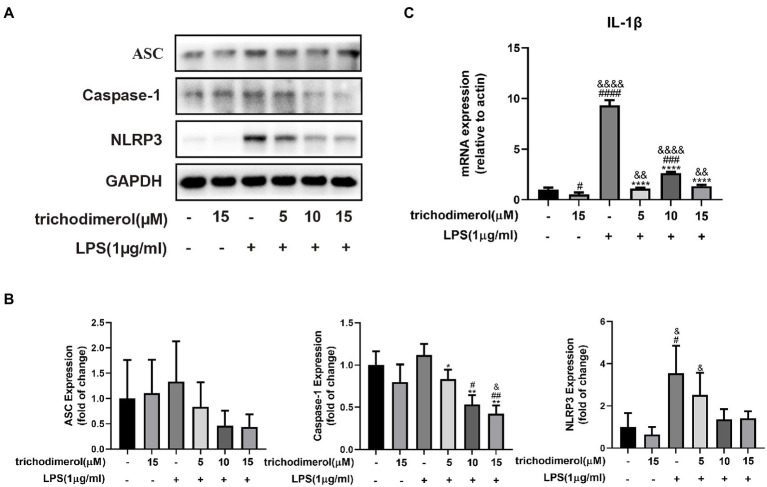
The effect of trichodimerol on the NLRP3 pathway. **(A)** RAW264.7 macrophages were treated with different concentrations of trichodimerol (5, 10, and 15 μM) for 2 h, followed by LPS (1 μg/ml) for 24 h. The protein expression of ASC, Caspase-1, and NLRP3 was detected by Western blotting; GAPDH was used as an internal reference. **(B)** Quantitative statistical results of the protein expression of ASC, Caspase-1, and NLRP3. **(C)** mRNA expression levels of IL-1β. All data are expressed as the mean ± SD. ^#^*p* < 0.05, ^##^*p* < 0.01, ^###^*p* < 0.001, ^####^*p* < 0.0001, compared with the DMSO group. ^&^*p* < 0.05, ^&&^*p* < 0.01, and ^&&&&^*p* < 0.0001, compared with the trichodimerol 15 μM group. ^*^p < 0.05, ^**^p < 0.01, and ^****^p < 0.0001, compared with the LPS group.

Multiple pathogen-and damage-associated stresses drive inflammation by activating the multimolecular NLRP3-inflammasome complex (Seoane et al., 2020), which is composed of ASC, Caspase-1, and NLRP3. To explore the effect of trichodimerol on the NLRP3 pathway, LPS was used to induce RAW264.7 macrophages. After trichodimerol administration, compared with LPS, the protein levels of ASC, Caspase-1, and NLRP3 were downregulated (Figures 5A,B), and the mRNA expression of IL-1β downstream was significantly downregulated (Figure 5C). This result indicated that the inflammatory response may be inhibited by restraining the NLRP3 pathway.

### Trichodimerol blocked the NF-κB pathway

**Figure 6:**
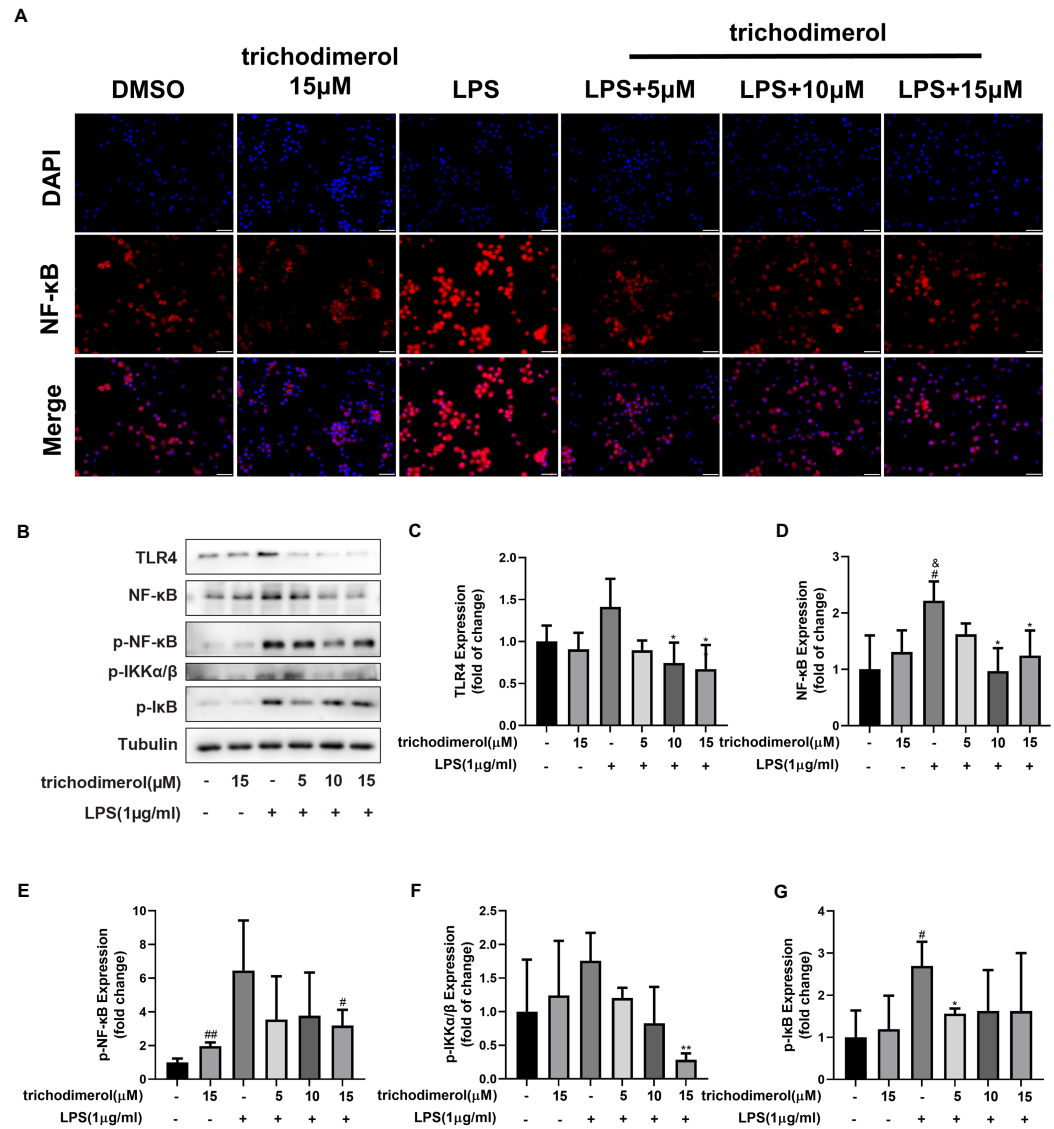
The effect of trichodimerol on the NF-κB pathway. **(A)** Nuclear translocation of RAW264.7 macrophages induced by LPS after 12 h of trichodimerol treatment. Red fluorescence represents the NF-κB signal, blue fluorescence represents the nucleus, white stripe = 50 μm. **(B)** The protein expression of TLR4, NF-κB, p-NF-κB, p-IKKα/β, and p-IκB in RAW264.7 macrophages treated with different concentrations; Tubulin was used as an internal reference. **(C)** Quantitative statistical results of TLR4 protein expression. **(D)** Quantitative statistical results of the protein expression of NF-κB. **(E)** Quantitative statistical results of the protein expression of p-NF-κB. **(F)** Quantitative statistical results of the protein expression of p-IKKα/β. **(G)** Quantitative statistical results of the protein expression of p-IκB. All data are expressed as the mean ± SD. ^#^*p* < 0.05, ^##^*p* < 0.01, compared with the DMSO group. ^&^*p* < 0.05, compared with the trichodimerol 15 μM group. ^*^p < 0.05, ^**^p < 0.01, compared with the LPS group.

Toll-like receptor (Toll) is an important component of the innate immune system and plays a significant role in inflammation. When TLR4 recognizes various microbial pathogens, it stimulates the activation of the NF-κB pathway (Kawai and Akira, 2007). The activation of NF-κB in macrophages can trigger the inflammatory cascade. IκBα promotes NF-κB to remain in the cytoplasm, is unable to enter the nucleus, and prevents its transcription. When IKK is activated, IκBα is phosphorylated and degraded by ubiquitination, releasing NF-κB and causing it to enter the nucleus (Baker et al., 2011). Compared with LPS-induced RAW264.7 macrophages, after administration of trichodimerol, fluorescence microscopy showed that NF-κB entered the nucleus decreased (red fluorescence; Figure 6A). At the same time, the protein levels of NF-κB, TLR4, p-IKK, p-IκB, and p-NF-κB showed a downward trend after adding trichodimerol (Figures 6B–G), which suggests that trichodimerol may inhibit inflammation by blocking the NF-κB pathway.

### Trichodimerol possessed affinity for TLR4-MD2

**Figure 7:**
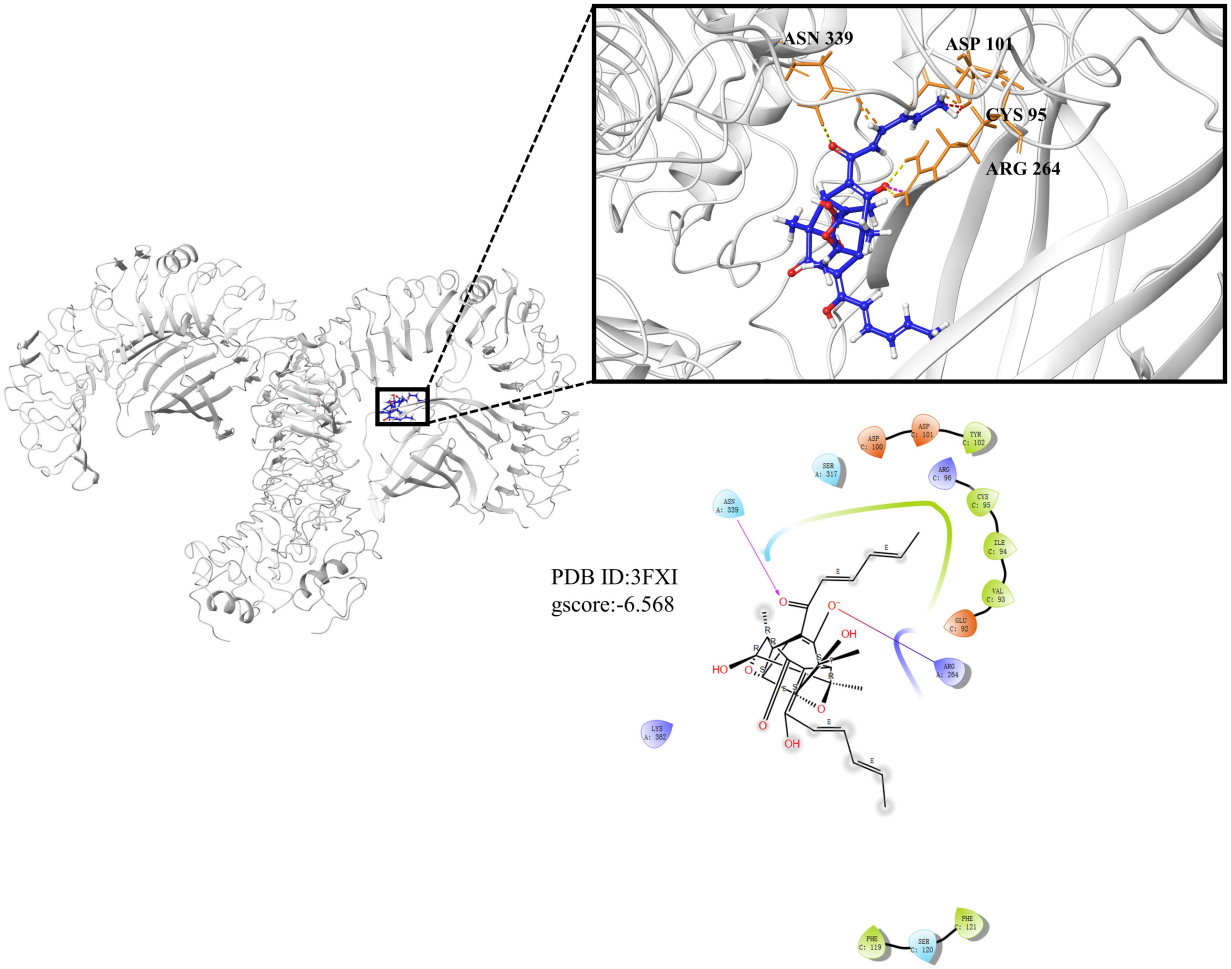
Molecular docking results of trichodimerol and TLR4-MD2. Molecular docking is simulated under the lowest energy conformation. Hydrogen bond interactions are represented by dotted lines.

TLR4-MD2 as the receptor of LPS is upstream molecule of NF-κB and plays an important role in inflammation, and molecular docking was used to predict whether TLR4 can competitively bind LPS with trichodimerol. The results showed that hydrogen bonds were formed between trichodimerol and four amino acid residues at the TLR4-MD2 active site, including CYS 95, ASP 101, ARG 264, and ASN 339 (Figure 7), which indicated that trichodimerol might have the ability to modify the conformation of the TLR4-MD2 complex and obstruct the interaction between LPS and the TLR4-MD2 heterodimer. Subsequently, downstream signaling pathways might be suppressed.

## Discussion

Acute inflammation is a self-defense protection produced by the human body that can subside by itself (Fredman et al., 2012). However, excessive injury time will further lead to the occurrence of chronic inflammation, such as asthma, arthritis, and cardiovascular diseases, which threaten human health (Zhong and Shi, 2019). Monocyte-derived macrophages play an active role in tissue regeneration and maintenance of tissue homeostasis (Viola et al., 2019). As an outer membrane component of Gram-negative bacteria, LPS plays an important role in inflammation (Batista et al., 2009). Induced by LPS, proinflammatory macrophages overexpress iNOS and COX-2 and produce a large number of proinflammatory cytokines, such as TNF-α, IL-1β, IL-6, and ROS (Reuter et al., 2010; Viola et al., 2019).

As a pattern recognition receptor of LPS, TLR4-MD2 plays a significant role in the development of inflammation. TLR4 receptor dimerization activates the downstream NF-κB and NLRP3 signaling pathways. The activation of NF-κB leads to the expression of proinflammatory factors, such as TNF-α, IL-1β, and IL-6 and upregulates the expression of NLRP3 (Bauernfeind et al., 2009; Rocha et al., 2016). Active NLRP3 oligomerizes and binds to the adaptor protein ASC (apoptosis-associated speck-like protein containing a caspase recruitment domain or CARD; Mezzaroma et al., 2021). Then, pro-caspase-1 is recruited and activated. Active caspase-1 cleaves its substrate pro-IL-1β to form mature IL-1β (White et al., 2017). IL-1β plays an important role in regulating the expression of adhesion molecules, mediating the inflammatory response, and immune cell infiltration (Wang et al., 1995). Based on this, we performed molecular docking of TLR4. The results showed that trichodimerol binds to TLR4-MD2 and forms hydrogen bonds with the four residues of the active site (Figure 7), suggesting that blocking TLR4-MD2 and suppressing TLR4-related downstream signaling pathways, such as the NF-κB (Figure 6) and NLRP3 (Figure 5) pathways, might be the underlying mechanism of trichodimerol’s anti-inflammatory activity. In this study, we confirmed that trichodimerol inhibited inflammation and partially uncovered a related mechanism. Further studies are needed to determine whether trichodimerol inhibits LPS binding to other sites and pathways.

## Conclusion

In this study, we used LPS-induced RAW264.7 macrophages and zebrafish to confirm that trichodimerol reduces the production of ROS *in vitro* and *in vivo*, NO, and the expression of proinflammatory factors, such as IL-6, TNF-α, COX-2, and iNOS and revealed that trichodimerol is anti-inflammatory through the NF-κB and NLRP3 pathways. Molecular docking was also applied to provide a possible interpretation. These results suggested that trichodimerol may become a hit compound for the treatment of inflammation.

## Data availability statement

The original contributions presented in the study are included in the article/Supplementary material; further inquiries can be directed to the corresponding authors.

## Ethics statement

The animal study was reviewed and approved by Ethics Committee of Chengdu University of Traditional Chinese Medicine.

## Author contributions

D-LG and YD designed and supervised the article. Q-XK performed the experiments and collected the data. X-YH, L-RL, and Y-JH analyzed and plotted the data. W-XG and M-DL isolated and purified trichodimerol. X-YH, L-RL, Y-JH, and Q-XK participated in the experiments. Y-FD and L-RL performed the molecular docking. X-YH wrote and finalized the manuscript. WP, Y-CG, D-LG, and YD contributed to the writing of this manuscript. All authors contributed to the article and approved the submitted version.

## Funding

This work was supported by the National Natural Science Foundation of China (U19A2011 and 81973460), Department of Science and Technology of Sichuan Province (2021ZYD0079 and 2021YFN0134), Chengdu University of Traditional Chinese Medicine (CZYJC1905, 2020XSGG016, and 2020JCRC006), and National Interdisciplinary Innovation Team of Traditional Chinese Medicine (ZYYCXTD-D-202209).

## Conflict of interest

The authors declare that the research was conducted in the absence of any commercial or financial relationships that could be construed as a potential conflict of interest.

## Publisher’s note

All claims expressed in this article are solely those of the authors and do not necessarily represent those of their affiliated organizations, or those of the publisher, the editors and the reviewers. Any product that may be evaluated in this article, or claim that may be made by its manufacturer, is not guaranteed or endorsed by the publisher.
