# Supplementary material for: Trichodimerol inhibits inflammation through suppression of the nuclear transcription factor-kappaB/NOD-like receptor thermal protein domain associated protein 3 signaling pathway
Source: Front Microbiol. 2022 Aug 23;13:999996. doi: 10.3389/fmicb.2022.999996 (PMC9445571; doi:10.3389/fmicb.2022.999996)
Supplement: Supplementary file 1 [file Data_Sheet_1.PDF]

Trichodimerol Inhibits Inflammation through Suppression of NF-κB/NLRP3 Signaling Pathway

Xue-Yan Huo[1,2,#], Li-Rong Lei[1,2,#], Wen-Xiu Guo[1,2], Yun-Jie Hu[1,2], Qi-Xuan Kuang[1,2], Meng-Dan Liu[1,2], Yi-Fei Dai[3], Dong Wang[1], Yu-Cheng Gu[4], Da-Le Guo[1,2*] and Yun Deng[1,2*]

[1] State Key Laboratory of Southwestern Chinese Medicine Resources, Chengdu University of Traditional Chinese Medicine, Chengdu 611137, People's Republic of China

[2] School of Pharmacy, Chengdu University of Traditional Chinese Medicine, Chengdu 611137, People's Republic of China

[3] Department of Basic Medical Sciences, School of Medicine, Tsinghua University, Beijing, 100084, People's Republic of China

[4.] Syngenta Jealott's Hill International Research Centre, Berkshire, U.K.

*** Correspondence:**
Corresponding Author
guodale@cdutcm.edu.cn (Da-Le Guo); dengyun@cdutcm.edu.cn (Yun Deng)

[#] Xue-Yan Huo and Li-Rong Lei contributed equally to this work.

1 All the raw data supporting the conclusions of this article will be made available at: https://www.jianguoyun.com/p/DbVp4N4Q1YPjChiY0swEIAA

2 Figures

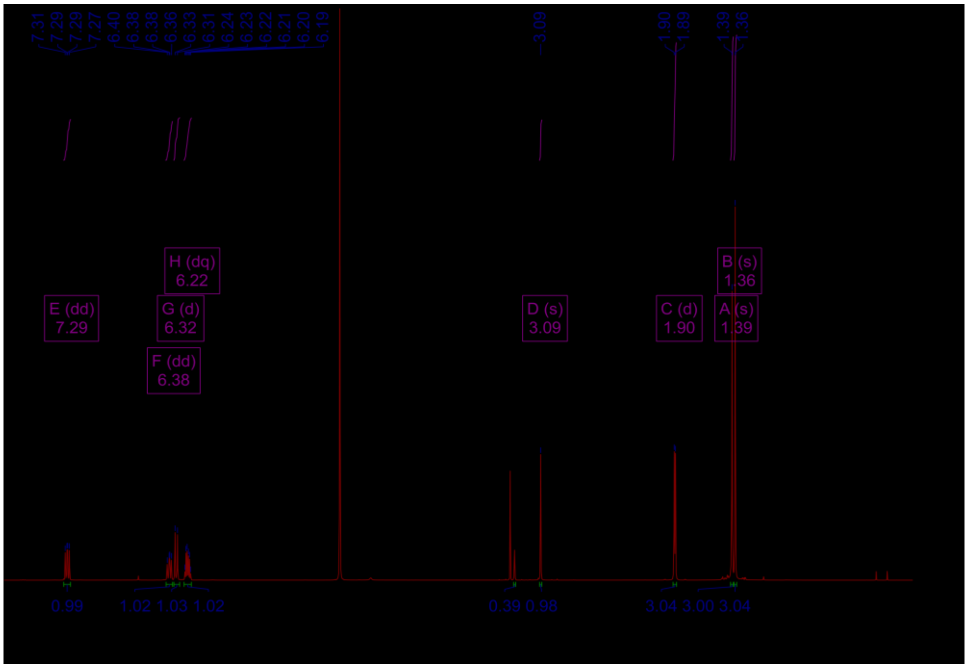

Figure S1： ^{1}H-NMR spectrum (700 MHz) of trichodimerol measured in CD_3OD.

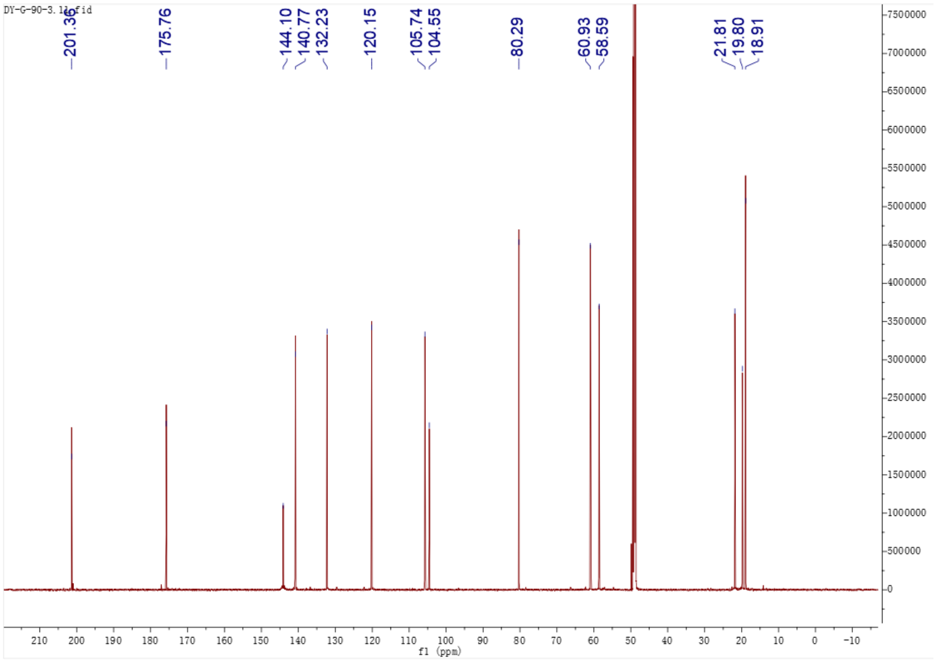

Figure S2.：^{13}C-NMR spectrum (175 MHz) of **trichodimerol** measured in CD3OD.

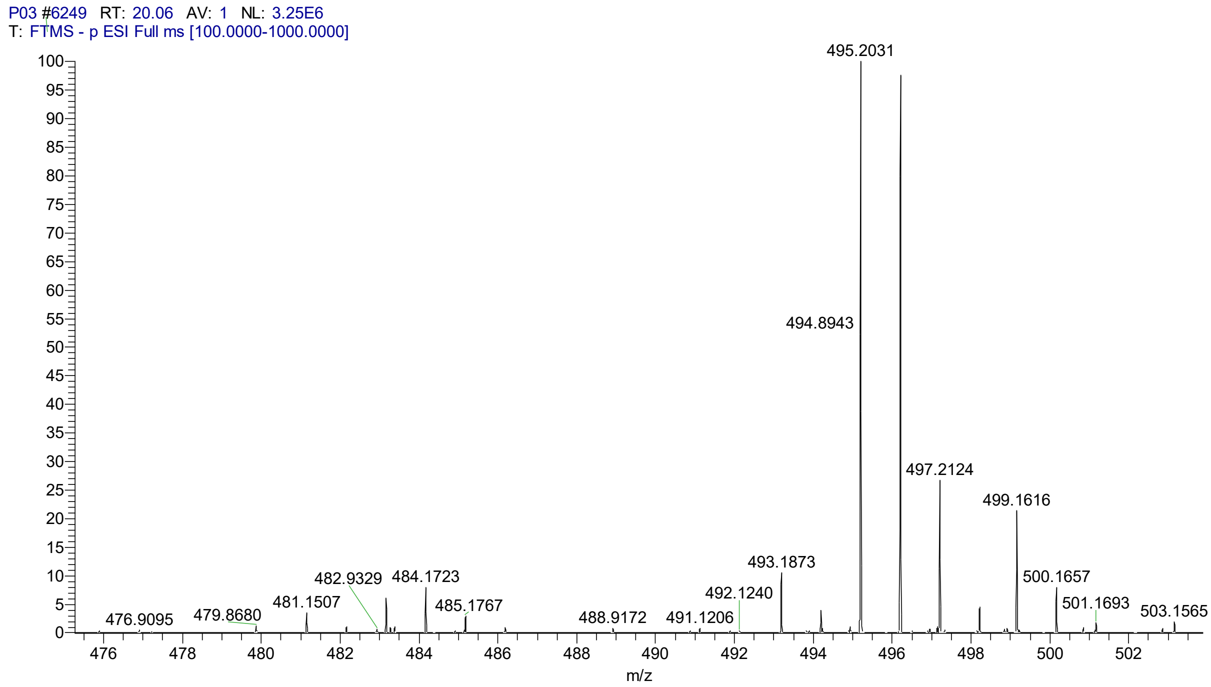

Figure S3.：The HRMS spectrum of compound trichodimerol.
